# Kinetics of Serum Myoglobin and Creatine Kinase Related to Exercise-Induced Muscle Damage and *ACTN3* Polymorphism in Military Paratroopers Under Intense Exercise

**DOI:** 10.3390/jfmk10040381

**Published:** 2025-10-02

**Authors:** Rachel de S. Augusto, Adrieli Dill, Eliezer Souza, Tatiana L. S. Nogueira, Diego V. Gomes, Jorge Paiva, Marcos Dornelas-Ribeiro, Caleb G. M. Santos

**Affiliations:** 1 Molecular Diagnostic Laboratory, Instituto de Biologia do Exército, Exército Brasileiro, Rio de Janeiro 20911-270, Brazil; racheldesousa@gmail.com (R.d.S.A.);; 2 Clinical Analyses Laboratory, Hospital Central do Exército, Exército Brasileiro, Rio de Janeiro 20911-270, Brazil; 3 Department of Running, School of Physical Education and Sport, Universidade Federal do Rio de Janeiro, Rio de Janeiro 20911-270, Brazil; 4 Teaching Division, Parachutist Instruction Center General Penha Brasil, Exército Brasileiro, Rio de Janeiro 20911-270, Brazil

**Keywords:** exercise-induced muscle damage, physical exercise, biomarkers, *ACTN3*, creatine kinase

## Abstract

**Background**: Physical conditioning is essential to meet the operational demands of military environments. However, high-intensity exercise provokes muscle microinjuries resulting in exercise-induced muscle damage. This condition is typically monitored using serum biomarkers such as creatine kinase (CK), myoglobin (MYO), and lactate dehydrogenase (LDH). Nevertheless, individual variability and genetic factors complicate the interpretation. In this context, the rs1815739 variant (*ACTN3*), the most common variant related to exercise phenotypes, hypothetically could interfere with the muscle physiological response. This study aimed to evaluate the kinetics of serum biomarkers during a high-intensity activity and their potential association with rs1815739 polymorphism. **Materials and Methods**: 32 male cadets were selected during the Army Paratrooper Course. Serum was obtained at six distinct moments while they performed regular course tests and recovery time. Borg scale was assessed in 2 moments (~11 and ~17). **Results**: Serum levels of CK, CK-MB, MYO, and LDH significantly increase after exercise, proportionally to Borg’s level, following the applicability of longitudinal studies to understand biomarker levels in response to exercise. R allele carriers (*ACTN3*) were only slightly associated with greater levels of MYO and CK, mainly in relative kinetic levels, and especially at moments of greater physical demand/recovery. Although the *ACTN3* was slightly related to different biomarker levels in our investigation, the success or healthiness in military activities is multifactorial and does not depend only on interindividual variability or physical capacity. **Conclusions**: Monitoring biomarkers and multiple genomic regions can generate more efficient exercise-related phenotype interventions.

## 1. Introduction

Training and good physical conditioning are necessary to meet military operational requirements, which routinely require a higher level of physical effort and vigor than most civilian occupations [1,2]. High-intensity exercise provokes muscle microinjuries resulting from biochemical events and mechanical overload, a normal response to exercise, or the physiological adaptation process [3]. However, more severe exercise-induced muscle damage (EIMD), such as exertional rhabdomyolysis (ER)—an ultrastructural damage to skeletal muscle with renal consequences—has great importance in military populations [4,5,6]. The incidence of EIMD is relevant in US soldiers, varying between 0.3% in Marine recruits, 3.0% for Army officer candidates, and around 2–40% in basic military training, leading to the cause of outpatient injuries among US soldiers [7,8,9].

EIMD is more prevalent in untrained individuals who perform high-intensity exercise under conditions of poor acclimatization, inadequate temperature, humidity, altitude, and hydration during exercise. Cardiometabolic, musculoskeletal, neuroimmunoendocrine, and infectious disorders, as well as the use of drugs and supplements, join genetics and inter-individual variations as key factors for damage [10,11,12]. Depending on the volume and intensity of the exercise, there is an increase in membrane permeability, allowing the extravasation of substances to the extracellular medium [13,14] such as the biomarkers myoglobin (MYO), creatine kinase (CK), and lactate dehydrogenase (LDH) [15,16,17].

Described as the most popular indirect biomarker for EIMD [13], the cytosolic form of CK has two subunits, M (muscular) and B (brain), forming three isoenzymes: MM, MB, and BB [18,19]. CK is especially abundant in skeletal muscle (CK-MM, 98%; CK-MB, 2%), cardiac muscle (CK-MM, ~75%; CK-MB, ~25%), and the brain (mostly CK-BB). Its serum discharge may indicate the transition from an adaptive microtrauma to a subclinical state between muscle fatigue and damage [20], or a clinical state if more extensive. CK levels for EIMD recognition are a topic of controversy. Values between 3 and 5 times the reference limit were common; however, their low specificity in athletes led to proposals for 10 times the reference value [10,21]. For military personnel training, the threshold for damage is routinely higher than for civilians, reaching values of even 50 times the reference levels [22].

Although the greater presence of CK-MB in the myocardium suggests some cardiac involvement, its elevation in serum may also reflect EIMD accompanying the increase in muscle CK, which reinforces its importance for studies evaluating the differential diagnosis of significant cardiac or muscle lesions [23,24,25]. In turn, cleared from plasma within 24 h after injury, MYO has a limited capacity to monitor microlesions over long periods, but has significant nephrotoxicity [10]. When its plasma release exceeds the binding/removal capacity, it accumulates and can progress to acute renal failure. [26,27]. Responsible for the interconversion of pyruvate and lactate, LDH can reflect not only cellular degradation but also the utilization of glycogen or even anaerobic capacity when meeting energy demands during resistance exercises [28,29]. Its slight increase, with peaks at 6 h, followed by a rapid return to basal levels, currently confers limitations on its use.

The individual variability observed during exercise makes interpreting traditional serum biomarkers challenging without accounting for genetics. In this context, the rs1815739 variant in the *ACTN3* gene, which encodes the alpha-actinin-3 protein, takes on significance, since this is the most extensively studied gene in exercise-related phenotypes. By anchoring thin filaments of actin to the Z-line in fast muscle fibers, alpha-actinin-3 primarily stabilizes the contractile apparatus, particularly in strength-power activities [30,31,32,33]. However, the nonsense variant in *ACTN3* (rs1815739), represented by the X allele, is characterized by the absence of the functional protein, leaving the role to the similar alpha-actinin-2 [34,35,36]. The presence of alpha-actinin-3 (R allele) was associated with better strength-power performances but yielded limited results in aerobic endurance exercise [37,38,39]. Although physiologically distinct, data regarding *ACTN3* genotypes, serum biomarker levels, and EIMD remain highly controversial [40,41,42]. Given the contextual panorama presented and considering that injuries cause a greater impact on the health of military troops on call [1], this study aimed to evaluate the biomarkers traditionally involved in muscle injury during intense physical activity and investigate their variability depending on the presence of polymorphic alleles in the *ACTN3* gene.

## 2. Materials and Methods

### 2.1. Ethical Approval and Sampling

All participants signed the Informed Consent Form. The study was approved by the Research Ethics Committee of the Army Physical Training Center—CCFEX (58413222.2.0000.9433) according to the Declaration of Helsinki (1964) on 28 June 2022. Thirty-two male cadets at the Agulhas Negras Military Academy (AMAN) were selected during the Basic Paratrooper Course. The inclusion criteria comprised a Cooper pre-test with at least 3000 m in 12 min and to be a final-year cadet. Participants with orthopedic injuries, muscle pain, who were using analgesics, anti-inflammatories, or supplements such as caffeine or thermogenics, were excluded. However, there was no restriction on water and food intake or sleep deprivation.

### 2.2. Biological Samples and Physical Activity

**Figure 1:**
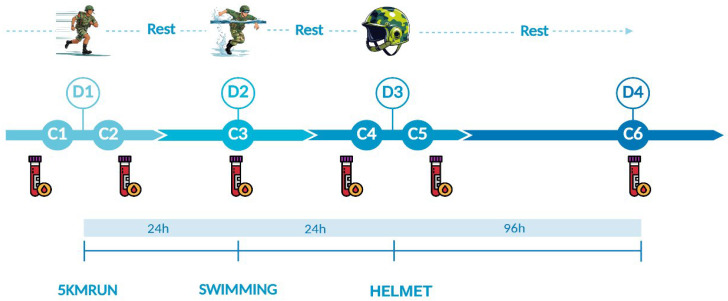
Experimental protocol. Day 1 (D1): collection before and after a 5 km run in up to 25 min, wearing camouflage pants and combat boots (C1 and C2, respectively). Day 2 (D2): collection (C3) 24 h after C1 and before free-time 4 m rope test, and swimming (50 m in 2 min and 30 s). Day 3 (D3): collection before (C4) and after (C5) the “Helmet Marking ceremony”. Day 4 (D4): sixth and last collection (C6) performed 96 h after C4.

Peripheral blood was obtained using vacuum system tubes (Greiner Bio-One, Kremsmünster, Austria) in 8 mL tubes without anticoagulant following Figure 1’s scheme on four days (D1 to D4) and six distinct extractions (C1 to C6) from the median cubital or basilic veins. All physical activities were coordinated by a trained Military Officer and supported by an emergency medical team. On the parachutist course day one, the main activity was a 5 km run up to 25 min, wearing camouflage pants and combat boots (5KMRUN). On day two, they performed a free-time rope test and swam 50 m in up to 2 min and 30 s. On the third day, they performed the Helmet Marking Ceremony (HELMET) activity. C1, C3, C4, and C6 were collected before sunrise at 4–5 a.m., and C2/C5 at 9–10 a.m.

The activity includes a ceremonial entry and exit of the course area, followed by Kangaroos, jumping jacks, and regular push-ups under the instructor’s command and between Throop’s displacements. On average, cadets performed approximately 420 four-count kangaroo jumps, 200 push-ups, and 150 jumping jacks (Table 1). The movement throughout the course area happens in military formation at a fast pace, and the movements are stereotyped: knees are exaggeratedly raised (elevation with excessive hip flexion), feet hit the ground firmly, heads remain raised, and the torso stays straight. They perform 3 × fifty-minute sections with 10 (ten) minute intervals, following Table 1 [43].

### 2.3. Rating of Perceived Exertion and Clinical Analysis

The Rating of Perceived Exertion (RPE) obeyed the Borg Scale protocol from 6 to 20 [36,44]. Level 6 is for resting time, and 20 is for maximal exertion. RPE was applied only after 5KMRUN (D1:C2) and after the HELMET (D3:C5) moments, individually and immediately after the activity, due to logistical and functional course limitations. Serum enzymatic activities of Total CK and LDH were analyzed using the photometric method, while the biomarkers MYO and CK-MB were analyzed using chemiluminescence. All analyses were performed using samples on the Alinity platform (Abbott Diagnostics, Abbott Park, IL, USA) [4].

### 2.4. The ACTN3 Polymorphism

The EDTA tubes containing peripheral blood samples were subjected to genomic DNA extraction using magnetic beads on the EXTRACTA 32 platform (Loccus, Cotia, Brazil). DNA obtained was verified by spectrophotometry using Nanodrop 2000c (Thermo Scientific, Waltham, MA, USA) and stored at −20 °C. SNP genotyping in the *ACTN3* gene (rs1815739) was performed by allelic discrimination assay using a validated commercial TaqMan assay for polymerase chain reaction (qPCR) with readings in the StepOnePlus Platform (Thermofisher, Foster City, CA, USA), using 10 ng of DNA input and TaqMan Genotyping Master Mix^®^ (2x) for a 10-microliter total volume assay (Thermofisher, Foster City, CA, USA). The genotype determination was performed using the StepOnePlus Software v2.3 and confirmed using the TaqMan Genotyper software v1.0 (Thermo Fisher, Foster City, CA, USA).

### 2.5. Statistical Analysis

The Kolmogorov–Smirnov test was used to evaluate the distribution of all data. RPE medians were compared using Wilcoxon tests for paired samples. Plasma concentrations of CK, MYO, LDH, and CK-MB were analyzed statistically using GraphPad Prism software v10. Parametric statistics (paired Student’s t-test) was applied to data on plasma levels with a normal distribution (LDH). Non-normally distributed data (CK, CK-MB, and MYO) were analyzed using non-parametric statistics, specifically the paired Wilcoxon tests for analyses at different time points (C1 and C6).

The minimal sample size was estimated based on the capacity of our approach to discriminate at least two essential moments for longitudinal analysis. Based on CK (the most common marker) variability and its non-normal distribution, we performed a Sample size adequacy assessment based on the Wilcoxon signed-rank test and observed effect size between C4 and C5 moments (effect size = 0.77). Thus, with a statistical power of 80% and a significance level of 5% in a two-tailed test, the sample size calculation (r = Z/√n) revealed that at least 14 participants would be needed to detect such an effect, for example, between C4 and C5 [45].

The genotype distribution for the rs1815739 polymorphism was tested for possible deviations from the Hardy–Weinberg equilibrium using the chi-square test. For genetic analysis, the study considered the population models of genotypes (RR, RX, and XX) and the recessive (RR+RX and XX) grouping of heterozygous and more frequent homozygous individuals in the same class based on their physiological phenotype. The nonparametric Kruskal–Wallis and Mann–Whitney tests were applied to assess biomarkers (CK, CK-MB, and MYO) according to the genotype and recessive models. For LDH and the same genetic models, parametric tests of one-way ANOVA and the t-test for independent samples were applied. The Bonferroni correction adjusted the statistical significance of multiple comparisons of exercise-related phenotypes. It takes into account the main independent variables of different biomarkers and Borg scale measures. The variation in biomarkers (Δ) was analyzed as Δ5KMRUN (C2-C1), ΔHELMET(C5-C4), and ΔRECOVER (C6-C5).

For MYO/CK and *ACTN3* associations with significant or marginal *p*-values, the effect size was calculated using Rosenthal’s “r” method for the genotype model, and Epsilon Squared (ε^2^) method for the recessive model. The confidence intervals for the median were estimated using bootstrap resampling with 1000 iterations, applying the percentile method.

## 3. Results

### 3.1. Exercise and Rating of Perceived Exertion

All participants completed the physical evaluations as requirements for the Basic Paratrooper Course. Due to the lower intensity and logistics, we cannot apply the subjective effort or post-activity blood sampling for the rope and swim (50 m in 2 min, 30 s) tests (D2). 5KMRUN and HELMET were used only as an eliminatory step. So, we used the Borg scale as a tool for the subjective evaluation of effort at the moments when it was possible to obtain it. The RPE analyses showed that the medians at C5 were very close to the maximum values of the scale and were significantly higher (*p* < 0.0001) than at C2, indicating greater intensity in the HELMET compared to 5KMRUN. (Table 2). Although exhausted at the end of the activity and with high RPE levels, no cadet presented ER.

### 3.2. The Laboratory Markers

**Figure 2:**
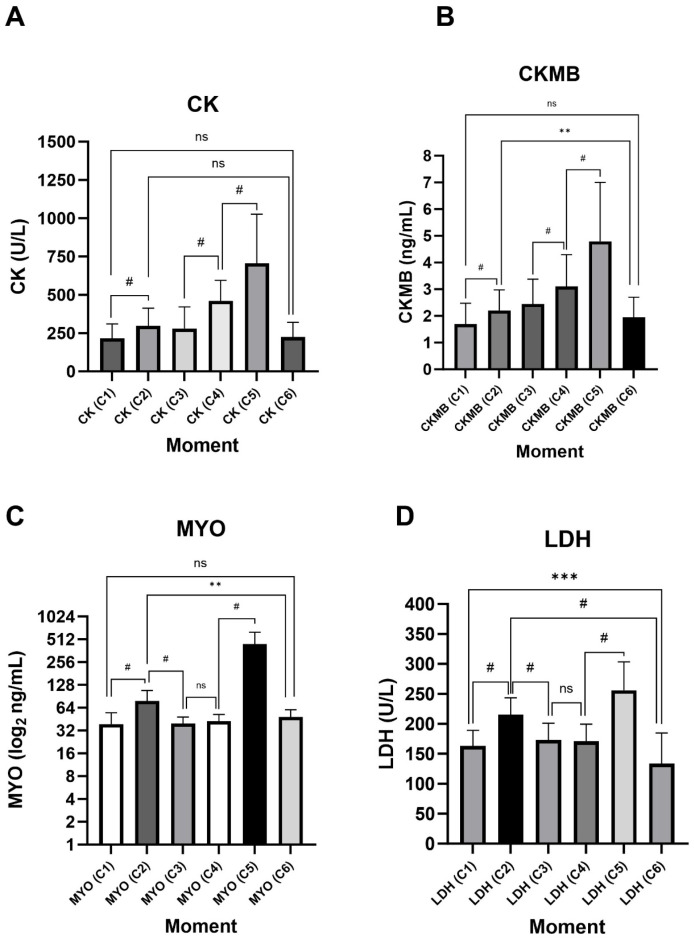
Biomarker serum levels in the study moments. Median by CK (**A**), CK-MB (**B**), MYO (**C**), and means by LDH (**D**) in C1, C2, C3, C4, C5, and C6 moments. ** *p* < 0.01; *** *p* < 0.001; ^#^ *p* < 0.0001; ns = Not significant.

Corroborating RPE results, all biomarkers showed a significant increase in their plasma concentrations in C2 and C5, with an even higher increase after HELMET (C5) (Figure 2). In turn, the medians at C6, for all biomarkers, were lower than in C2 or C5, but not different from the medians at baseline C1, pointing to a satisfactory muscle recovery of the participants. CK and CK-MB levels were very similar during all time points, except that CK-MB levels after 5KMRUN were significantly higher than C6. Both markers were increased before HELMET, compared to C3, showing that this increase may result from accumulating the previous day’s activities. Similarly, LDH means 96 h after HELMET (C6) was lower than those on C1, probably because some non-specific activity occurred before the course began. The MYO graph presented results plotted on a log2 scale to better allow the visualization of differences in plasma concentrations. However, clearly, the variation concentrated itself after immediately exercising, pointing to MYO’s faster turnover.

### 3.3. ACTN3 Gene SNP Analysis

The *ACTN3* (rs1815739) genotype distribution showed a higher frequency for the CT genotype (RX), followed by the CC genotype (RR) and, less frequently, the TT genotype (XX) according to the Hardy–Weinberg principle (*p* = 0.9306, R: 0.6094, X: 0.3906, and Chi-squared Value: 0.0076). Additionally, Cooper’s previous tests and RPE were not different among different groups on genotypes or recessive genetic models (Table 3).

Incorporating the genotype groups into the biomarkers analysis, it was observed that the different intramarker medians/means were not systematically influenced by the *ACTN3* genotypes or by the presence of at least one of the R alleles (Table 4). The highest absolute values in all groups remained at C5 (peak of exercise), followed by a reduction in serum levels to values comparable to the initial values at C6 (Table 4). Table 4 shows only some trends involving genetic differences, which show CK levels at C5, (*p* = 0.090/ES = 0.097 and *p* = 0.077/ES = 0.320), and MYO levels in C1 and C2, respectively (*p* = 0.087/ES = 0.310, *p* = 0.068/ES = 0.326). At the same time, MYO was significantly more present in individuals with the presence of at least one R allele in the recessive model (*p* = 0.019/ES = 0.409) but only close to significance in the genotype model (*p* = 0.057/ES = 0.129). In turn, in evaluating variations (Δ) summarized in Table 5, although we found some marginal significance, the same biomarkers showed interesting results. the highest variation in MYO have been seen in participants with at least one R allele in ΔHELMET (*p* = 0.019). This difference extended to a more intense MYO ΔRECOVER in the same group (*p* = 0.016) of this same biomarker. Regarding CK, its variation (ΔRECOVER) was significantly higher in carriers of the R allele, both in the genotype model (*p* = 0.042) and the recessive model (*p* = 0.009).

## 4. Discussion

RPE represents how hard an individual feels their body is working, based on their perception of sensations that occur during physical activity. Although subjective, this effort rating can provide a reasonably good estimate of the level of physical demand [46]. The RPE of the “HELMET” moment, close to level 17, indicates a very intense effort, previously comparable, for example, to athletes trained after a Triathlon competition [41]; an effort that justifies the presentation of high blood levels of muscle injury markers. In turn, RPE on 5KMRUN (11) was comparable to 30 min of treadmill running with a 15% inclination, 70% VO_2max_ in recreational runners [36]. Although the activity was real, it is worth noting however, that the exercise protocols chosen in the present study are not representative of any specific sport, modality, type of exercise, or physical valence, a fact that made it difficult to compare the results with the literature since it is well known that specific tasks for military personnel involve the need for aerobic and anaerobic resistance and muscular strength to meet occupational demands [47].

It is well known that interindividual variability affects the magnitude of the response of biomarkers to injury. Factors such as gender, age, ethnicity, environmental conditions, physical fitness, muscle mass, and genetic predisposition are paramount for the response [48,49]. Because of this, it is justified to choose cadets of the same sex, class, with similar ages and training conditions, in order to minimize external effects. It is important to highlight that, although a Borg level 17 was reached, no cadet developed ER. In addition, it is essential to note that there are no markers considered the “gold standard” for EIMD or ER, its most severe consequence [50], and therefore, it is necessary to advance the study of biomarkers.

CK and MYO serum levels are considered the primary markers of muscle tissue functionality, increasing after strenuous physical activity [11,51]. LDH provides additional information, including biochemical adaptation to physical load [52,53]. In turn, CK-MB is predictive of cardiac injury but is increased during the repair of injured skeletal muscle via satellite cells [54]. Consistent with the literature, our results showed a significant increase in CK, MYO, LDH, and CK-MB values after physical activities, particularly after HELMET. American recruits [22] who underwent 3 days of basic military training (including running, squats, push-ups, sit-ups, and pull-ups) presented CK levels comparable to those of our participants in C1 and C5 moments, with mean values of approximately 220 U/L and 700 U/L, respectively. Similarly to our 5KMRUN, active street runners showed CK levels between 211 U/L and 295 U/L before and after a 6 km run, respectively, in approximately 33 min [55].

Consecutive physical activities could have affected the kinetics of biomarkers due to the accumulation of activities. The speed of their release and elimination in serum depends on the level of training and adaptation, physical fitness, type, intensity, and duration of the individual’s exercise. Thus, the timepoints of this study, obtained for convenience, were adjusted so as not to compromise the activities but, at the same time, to meet the kinetics of the already known markers (CK, MYO, and LDH), until their return to baseline values [23,56,57]. Park and colleagues [4] evaluated the same four markers in amateur triathlon athletes. The highest amplitude was observed in MYO, 13 times above baseline, comparable to 10 times above baseline for C5 (after HELMET) time point. Nevertheless, marathoners presented MYO levels higher than our population in all moments evaluated (24, 48, or 72 h) after running. Regarding LDH, the increase was 30% among triathletes and 50% among military personnel (after HELMET). On the other hand, the increase in CK and CK-MB levels after activity was considerably higher in triathletes. In prolonged exercise, for example, serum CK activity is markedly elevated after 24 h and can remain increased for 48 to 72 h, returning to baseline levels between 6 and 10 days [17,26]. In our study, the highest CK values were obtained at C5, returning to their basal levels 96 h after “HELMET”. However, we cannot be certain whether the accumulation of activities has shifted the peak of the markers between C5 and C6, and this was a significant limitation in timepoints.

While CK has a half-life of around 1.5 days, MYO has a half-life of 2 to 4 h, normalizing 6 to 8 h after muscle injury [10]. The MYO medians obtained in this study also did not remain increased in C3, much less on C6, indicating its total clearance from the bloodstream, despite the higher levels observed in C5 compared to C4, according to the greater intensity of HELMET about 5KMRUN. Regarding the CK-MB isoenzyme, it is essential to highlight its wide expression in cardiac and skeletal muscle. Therefore, CK-MB values must be interpreted carefully as they can report skeletal and muscular involvement simultaneously, showing that CK-MB is not myocardium-specific after strenuous exercise. After intense exercise, some reports have shown that the CK-MB isoenzyme contributes 1–19% of CK levels; therefore, it is not surprising that its kinetics in response to exercise may be similar [11,58]. Our CK and CK-MB serum median variations corroborate the findings of the literature, changing by 37% and 30% after 5KMRUN, respectively, and by 55% after HELMET [11,58]. Despite this, it is worth noting that CK was measured by enzymatic activity (U/L), whereas CK-MB was measured by mass (ng/mL) using a chemiluminescent immunoassay. Although related, their magnitudes are distinct, and the direct comparison may have an important limitation in response to exercise.

LDH serum levels usually show a modest peak (20–30% above baseline) within 3–6 h post-exercise, returning to baseline after 24 h [51]. Our results showed variations of 32% and 50%, respectively, for C1-C2 and C5-C4, in addition to returning to baseline levels in C3 and C6, which is more pronounced than in triathletes previously described (1.5 km swimming, 40 km cycling, and 10 km running) [25].

**Figure 3:**
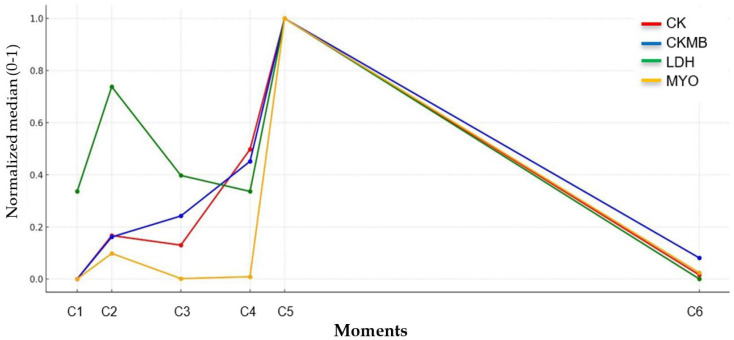
Relative comparative biomarker analysis. Kinetics analysis and relative comparison between classical biomarkers based on median values (and mean for LDH), normalized on a scale of 0 to 1. Descriptive dispersion measures were not presented, but they are available for absolute data in the Supplemental Table S1.

The kinetics analysis of classical biomarkers, evaluated based on median values (and mean for LDH), normalized on a scale of 0 to 1, allowed for a relative comparison between them. The similarity of the kinetic profiles between C5 and C6 (Figure 3), despite the different physiological half-lives, suggests that the collections were effective in indicating the recovery of the military personnel to levels close to baseline, but could not identify individual profiles of the markers after HELMET. Still, between C2 and C3, the shorter half-lives of LDH and MYO make it challenging to demonstrate injury in shorter rest periods; however, CK and CK-MB ultimately illustrate the need for a longer recovery time.

Although there appeared to have been no significant impact on D2 military activities (suggested by MYO and LDH, markers with faster depuration), the missing RPE values in D2 activities after C3 did not give us the opportunity to confirm it. The rs1815739 polymorphism in our participants had an allelic frequency compatible with that found in studies conducted in a European population [59,60,61], Brazilian soccer athletes [37,40,62], and with the world database 1000 genomes. However, a few more RR genotypes were observed, likely due to the presence of African populations’ alleles resulting from the miscegenation of our population (Table 6).

Known as the “speed gene”, rs1815739 is a non-pathogenic nonsense variant of the *ACTN3* gene, whose homozygous condition for the X allele results in the total absence of the alpha-actinin-3 protein in muscle fibers. This condition, prevalent in a large portion of the world’s population, has been linked to interindividual variability in physical performance [38,63,64]. The absence of this protein, which is crucial for contraction and essential in type II fibers, underscores the physiological relevance of the investigation [65]. Although our analysis did not identify systematic differences in biomarkers between *ACTN3* rs1815739 genotypes, we observed, after the HELMET protocol, higher absolute and relative levels of MYO in carriers of the R allele, corroborating results in marathon runners [66] but diverging from other studies with marathon runners and ultra-endurance athletes [41,67]. MYO is highly abundant and functional in skeletal muscle, and during exercise-induced microinjuries, it will undoubtedly be more available in the blood [50].

Regarding CK, the literature shows divergent results. Some studies indicate higher concentrations in RR/RX individuals, suggesting greater susceptibility to muscle damage [13,36,68]. Others, however, report elevations in carriers of the X allele [31,41,62,69], which would indicate greater muscle damage due to the absence of alpha-actinin-3. In our analysis, CK was higher in alphaactinin-3 carriers, but only in ΔRECOVER, indicating that CK kinetics are more relevant than absolute values (Table 5).

Multifactorial traits such as exercise-related phenotypes are challenging to understand because of the complex interplay of genetic predispositions and environmental exposures. An increasingly reliable measurement of those phenotypes, increased by biomarker kinetics understanding and genetics/other omics approaches, can favor not only military personnel but also athletes who perform intense activities, which demand optimal recovery time, volume monitoring, injury prevention and personalized training/nutritional interventions.

Despite satisfactory longitudinal biomarker analysis, genotype categorization significantly reduced degrees of freedom, especially for the XX genotype (n = 5), limiting power for genotype subgroup analyses in our genetic approach. Muscle injury is complex, with multiple variants contributing to the final phenotype, and SNPs individually explain the effect in a limited way, which may underestimate the polygenic complexity of muscle damage responses and recovery [70]. Although rs1815739 is a SNP candidate with an excellent physiological hypothesis, analyses of multiple variants in larger populations may bring more consistency to the results obtained [4,63]. Lastly, our approach controlled several confounders such as age, training status, and sex, but did not account for or standardize for potential influences from hydration status, libitum hydration/feeding/sleep levels, ambient temperature/humidity, circadian variation, or prior subclinical muscle injuries.

## 5. Conclusions

The longitudinal 6-point results obtained by the study involving classical serum biomarkers in a unique military population resulted in interesting results. However, the presence of more collection points during recovery time may be beneficial to identify kinetic patterns of recovery among biomarkers.

The genotypic distribution of *ACTN3* in the population evaluated reflects an admixed population. Its alleles were not markedly associated with traditional EIMD biomarkers in their absolute values, but its variability showed that R allele carriers were associated with CK and MYO levels, especially at moments of greater physical demand. However, the genetically categorized sample was reduced, especially for the XX genotype, which partially compromised the analysis of associations between the categories analyzed.

Success or healthiness in military activities is a multifactorial trait and does not depend only on interindividual variability or physical capacity. Monitoring biomarkers and a single genetic variant should be performed with caution, recognizing the need for more specific markers and multiple genomic regions to obtain efficient interventions.

## Figures and Tables

**Table 1:** Summary of activities carried out by the military personnel of the course.

Day	Duration	Activity
D1	25 min	- 5 km run in 25 min, with camouflage pants and combat boots
D2	2.5 min Free	- 50 m of swimming - 4 m of rope climb test without feet
D3	50 min	- 5 × 400 m running routines - 100 kangaroo flexions between running routines - 10 min break
	50 min	- 2 × 400 m running routines - 20 kangaroo flexions followed by 10 push-ups in 3 min - Interspersed, 150 kangaroo flexions and 150 jumping jacks in sets of 10 repetitions in 25 min - 10 min break
	50 min	- 150 kangaroo push-ups were performed interspersed with 200 regular push-ups in sets of 10 exercises

**Table 2:** Age and perceived exertion using the Borg scale (n = 32).

Variable	Median
Age	23.0 ± 2.0
Borg (C2)	11.0 ± 2.0
Borg (C5)	16.5 ± 3.0 **

** *p* < 0.01 Compared to C2. Data is presented as median ± interquartile range.

**Table 3:** Distribution of the genotypes/alleles of the *ACTN3* SNP as a function of the medians of the last Cooper’s test of the cadets and their RPE after the 5KMRUN (C2) and HELMET (C5).

Biomarker	TT (XX)	CT (RX)	CC (RR)	*p*	Allele R	Allele X	*p*
Frequency “n” (%)	5 (15.6)	15 (46.9)	12 (37.5)	0.085	27 (84.4)	5 (15.6)	<0.001 *
Last Cooper’s test (m)	3115 ± 158	3262 ± 181	3200 ± 150	0.080	3200 ± 150	3115 ± 158	0.070
RPE POST 5KMRUN (C2)	11.5± 2.0	11.0± 2.0	11.0± 5.0	0.940	11.0± 3.0	11.5± 2.0	0.9352
RPE POST HELMET (C5)	15.0± 3.0	16.0± 3.0	17.0± 3.0	0.514	16.5± 3.0	15.0± 3.0	0.620

Note: Values are presented as frequencies or median ± interquartile range. * Chi-square test for genotypes.

**Table 4:** Medians/means of the genotypes and alleles of the *ACTN3* gene as a function of the CK, CK-MB, MYO, and LDH enzymes.

BIOMARKER	All (n = 32)	TT (XX)	CT (RX)	CC (RR)	*p* (Genotype Model)	RR + RX	XX	*p* (Recessive Model)
CK (C1)	217.0 ± 139.0	175.0 ± 62.0	217.0 ± 117.0	244.5 ± 112.0	0.284	228.0 ± 141.0	175.0 ± 62.0	0.166
CK (C2)	298.5 ± 181.0	257.0 ± 76.0	298.0 ± 195.0	324.5 ± 192.0	0.249	324.0 ± 177.0	257.0 ± 76.0	0.109
CK (C3)	280.5 ± 196.0	243.0 ± 114.0	277.0 ± 214.0	349.5 ± 242.0	0.310	283.0 ± 202.0	243.0 ± 114.0	0.263
CK (C4)	460.0 ± 221.0	422.0 ± 206.0	442.0 ± 172.0	498.0 ± 312.0	0.164	465.0 ± 211.0	422.0 ± 206.0	0.310
CK (C5)	705.5 ± 421.0	598.0 ± 299.0	708.0 ± 290.0	848.5 ± 445.0	0.090 (ES = 0.097)	711.0 ± 451.0	598.0 ± 299.0	0.077 (ES = 0.320)
CK (C6)	224.5 ± 163.0	208.0 ± 1315.0	229.0 ± 175.0	235.0 ± 139.0	0.681	229.0 ± 145.0	208.0 ± 1315.0	0.999
CK-MB (C1)	1.7 ± 1.1	2.2 ± 1.1	1.7 ± 0.7	1.6 ± 1.2	0.694	1.6 ± 1.0	2.2 ± 1.0	0.448
CK-MB (C2)	2.2 ± 1.1	2.9 ± 1.1	2.0 ± 1.0	2.1 ± 1.9	0.771	2.0 ± 1.0	2.9 ± 1.0	0.545
CK-MB (C3)	2.4 ± 1.6	3.0 ± 2.2	2.3 ± 1.5	2.4 ± 2.1	0.558	2.3 ± 2.0	3.0 ± 2.0	0.545
CK-MB (C4)	3.1 ± 2.0	4.3 ± 3.5	3.0 ± 2.0	3.1 ± 1.8	0.274	3.0 ± 2.0	4.3 ± 3.0	0.285
CK-MB (C5)	4.8 ± 3.3	5.9 ± 3.7	4.5 ± 2.8	4.5 ± 6.7	0.893	4.5 ± 4.0	5.9 ± 4.0	0.658
CK-MB (C6)	1.9 ± 1.5	2.3 ± 1.9	1.6 ± 0.9	2.1 ± 1.5	0.171	1.6 ± 1.0	2.3 ± 2.0	0.999
MYO (C1)	38.9 ± 25.1	28.6 ± 26.6	39.6 ± 25.6	39.5 ± 31.8	0.220	39.6 ± 22.0	28.6 ± 27.0	0.087 (ES = 0.310)
MYO (C2)	78.4 ± 46.0	66.1 ± 22.8	79.4 ± 42.0	94.4 ± 57.8	0.170	79.7 ± 56.0	66.1 ± 23.0	0.068 (ES = 0.326)
MYO (C3)	39.6 ± 13.5	34.7 ± 18.2	40.0 ± 9.2	39.8 ± 18.8	0.473	40.5 ± 15.0	34.7 ± 18.0	0.241
MYO (C4)	42.4 ± 14.2	40.9 ± 10.2	45.6 ± 15.5	40.7 ± 13.4	0.627	44.9 ± 16.0	40.9 ± 10.0	0.650
MYO (C5)	441.3 ± 359.1	228.3 ± 215.1	457.8 ± 351.5	497.3 ± 379.2	0.057 (ES = 0.129)	489.1 ± 345.0	228.3 ± 215.0	0.019 (ES = 0.409)
MYO (C6)	48.5 ± 23.4	41.4 ± 99.5	46.5 ± 19.6	50.9 ± 36.8	0.910	49.9 ± 21.0	41.4 ± 100.0	0.880
LDH (C1)	166.0 ± 38.0	164.2 ± 73.0	157.4 ± 24.0	170.5 ± 37.0	0.421	163.2 ± 33.0	164.2 ± 73.0	0.954
LDH (C2)	210.0 ± 36.0	222.6 ± 78.0	202.5 ± 31.0	225.7 ± 47.0	0.126	211.4 ± 33.0	222.6 ± 78.0	0.676
LDH (C3)	174.0 ± 40.0	179.4 ± 78.0	169.0 ± 41.0	177.2 ± 26.0	0.600	172.4 ± 37.0	179.4 ± 78.0	0.713
LDH (C4)	166.0 ± 33.0	177.8 ± 73.0	162.4 ± 38.0	178.6 ± 36.0	0.297	169.6 ± 34.0	177.8 ± 73.0	0.669
LDH (C5)	240.0 ± 80.0	243.0 ± 95.0	242.5 ± 51.0	278.9 ± 82.0	0.109	258.1 ± 83.0	243.0 ± 95.0	0.590
LDH (C6)	127.0 ± 43.0	152.6 ± 140.0	115.3 ± 66.0	148.0 ± 41.0	0.248	130.5 ± 41.0	152.6 ± 150.0	0.796

Note: Data highlighted in blue represent the moments in which the markers presented values close to being considered statistically different as a function of the genotypes/alleles of *ACTN3*. In orange, the only time/biomarker in which statistically different values were verified as a function of the presence of the R allele is highlighted. The effect size (ES) was calculated in RR &RX&XX by Rosenthal’s “r” method and in RR& RX + XX by Epsilon Squared (ε^2^) method showing medium effects for all highlighted table cells.

**Table 5:** Variation (Δ) of the medians (CK, CK-MB, and MYO) and means (LDH) at different times: 5KMRUN (C2-C1), ΔHELMET (C5-C4), Δ RECOVER (C6-C5), categorized into groups by the genotype and recessive models of the *ACTN3* gene.

BIOMARKER	All (n = 32)	TT (XX)	CT (RX)	CC (RR)	*p* (Genotype Model)	RR + RX	XX	*p* (Recessive Model)
CK Δ5KMRUN	70.5 ± 54.0	59.0 ± 25.0	76.0 ± 78.0	73.0 ± 49.0	0.205	73.0 ± 58.0	59.0 ± 25.0	0.077
CK ΔHELMET	274.5 ± 222.0	235.0 ± 162.0	330.0 ± 214.0	261.0 ± 380.0	0.241	306.0 ± 314.0	235.0 ± 162.0	0.098
CK ΔRECOVER	476.0 ± 286.0	371.0 ± 1026.0	525.0 ± 224.0	483.0 ± 439.0	0.042	505.0 ± 348.0	371.0 ± 1026.0	0.009
CK-MB Δ5KMRUN	0.5 ± 0.5	0.6 ± 0.0	0.4 ± 0.0	0.5 ± 1.0	0.951	0.5 ± 1.0	0.6 ± 0.0	0.960
CK-MB ΔHELMET	1.7 ± 1.7	1.5 ± 2.0	1.9 ± 1.0	1.4 ± 3.0	0.648	1.7 ± 2.0	1.5 ± 2.0	0.696
CK-MB ΔRECOVER	3.3 ± 2.3	3.6 ± 2.0	3.0 ± 2.0	3.2 ± 4.0	0.915	3.2 ± 3.0	3.6 ± 2.0	0.793
MYO Δ5KMRUN	38.7 ± 30.9	30.1 ± 34.0	38.4 ± 27.0	39.4 ± 56.0	0.397	39.4 ± 40.0	30.1 ± 34.0	0.241
MYO ΔHELMET	390.1 ± 349.8	187.8 ± 205.0	412.6 ± 322.0	492.2 ± 410.0	0.056	436.8 ± 339.0	187.8 ± 205.0	0.019
MYO ΔRECOVER	370.5 ± 356.8	191.0 ± 186.0	417.5 ± 327.0	391.0 ± 408.0	0.057	402.6 ± 341.0	191.0 ± 186.0	0.016
LDH Δ5KMRUN	49.9 ± 20.1	58.4 ± 27.0	45.1 ± 27.0	52.0 ± 37.0	0.504	50.9 ± 20.0	58.4 ± 27.0	0.380
LDH ΔHELMET	84.6 ± 33.5	65.2 ± 64.0	80.0 ± 33.0	99.2 ± 63.0	0.089	89.1 ± 48.0	65.2 ± 64.0	0.218
LDH ΔRECOVER	123.3 ± 45.8	102.5 ± 83.0	127.2 ± 93.0	127.9 ± 57.0	0.499	128.9 ± 85.0	102.5 ± 83.0	0.271

Note: data in blue represent the moments when the markers presented Δ values close to being considered statistically different as a function of the genotypes/alleles of *ACTN3*. The moments in which the single biomarker (MYO) presented statistically different values due to the presence of the R allele are highlighted in orange.

**Table 6:** Frequency of the rs1815739 polymorphism genotypes in the *ACTN3* gene in the populations of the 1000 Genomes database.

Population	Total		
	RR	RX	XX
Military participants	37.5%	46.9%	15.6%
European	31.0%	51.1%	17.9%
Africans	77.5%	22.1%	0.4%
American	16.1%	53.3%	30.5%
East Asian	30.8%	49.8%	19.4%
South Asian	15.7%	51.1%	33.1%

## Data Availability

All data regarding serum biomarkers and genotypes for all research time points are available upon simple request.

## Supplementary Materials

The following supporting information can be downloaded at: https://www.mdpi.com/article/10.3390/jfmk10040381/s1, This material includes an .xls file with the complete data from the study.
